# Association between Public Reporting of Outcomes and the Use of Mechanical Circulatory Support in Patients with Cardiogenic Shock

**DOI:** 10.1155/2019/3276521

**Published:** 2019-09-15

**Authors:** Vikas Singh, Rodrigo Mendirichaga, Parth Bhatt, Ghanshyambhai Savani, Anil K. Jonnalagadda, Igor Palacios, Mauricio G. Cohen, William W. O'Neill

**Affiliations:** ^1^University of Louisville Medical School, Louisville, KY, USA; ^2^Boston Medical Center, Boston University School of Medicine, Boston, MA, USA; ^3^Texas Tech University Health Sciences Center, Amarillo, TX, USA; ^4^Baystate Medical Center, University of Massachusetts, Springfield, MA, USA; ^5^Medstar Washington Hospital Center, Washington, DC, USA; ^6^Massachusetts General Hospital, Harvard Medical School, Boston, MA, USA; ^7^University of Miami Miller School of Medicine, Miami, FL, USA; ^8^Henry Ford Hospital, Detroit, MI, USA

## Abstract

Risk-averse behavior has been reported among physicians and facilities treating cardiogenic shock in states with public reporting. Our objective was to evaluate if public reporting leads to a lower use of mechanical circulatory support in cardiogenic shock. We conducted a retrospective study with the use of the National Inpatient Sample from 2005 to 2011. Hospitalizations of patients ≥18 years old with a diagnosis of cardiogenic shock were included. A regional comparison was performed to identify differences between reporting and nonreporting states. The main outcome of interest was the use of mechanical circulatory support. A total of 13043 hospitalizations for cardiogenic shock were identified of which 9664 occurred in reporting and 3379 in nonreporting states (age 69.9 ± 0.4 years, 56.8% men). Use of mechanical circulatory support was 32.8% in this high-risk population. Odds of receiving mechanical circulatory support were lower (OR 0.50; 95% CI 0.43–0.57; *p* < 0.01) and in-hospital mortality higher (OR 1.19; 95% CI 1.06–1.34; *p* < 0.01) in reporting states. Use of mechanical circulatory support was also lower in the subgroup of patients with acute myocardial infarction and cardiogenic shock in reporting states (OR 0.61; 95% CI 0.51–0.72; *p* < 0.01). In conclusion, patients with cardiogenic shock in reporting states are less likely to receive mechanical circulatory support than patients in nonreporting states.

## 1. Introduction

Public reporting of outcomes was developed to improve transparency in healthcare, patient safety, and quality of care. States that have adopted some form of public reporting in cardiovascular care include Massachusetts, New Jersey, New York, Pennsylvania, and Washington [1, 2]. Unintended consequences including risk-averse behavior among physicians and facilities have emerged [3–5]. This has been particularly true for patients presenting with acute myocardial infarction (AMI) complicated by cardiogenic shock (CS) who are less likely to receive percutaneous coronary intervention (PCI) in reporting states [6, 7]. Despite advances in management, in-hospital mortality of CS remains high [8]. Efforts to reduce mortality have focused primarily on early reperfusion in patients presenting with AMI and use of mechanical circulatory support (MCS) [9, 10]. The objective of this study is to analyze whether the use of public reporting leads to a lower use of MCS in CS.

## 2. Materials and Methods

The study was conducted between January 2018 and May 2018 with the use of the National Inpatient Sample (NIS), Healthcare Cost and Utilization Project, Agency for Healthcare Research and Quality. The NIS is the largest publicly available database on all-payer administrative claims in the United States and has been extensively used to analyze trends in cardiovascular care [11, 12]. NIS data from January 2005 through December 2011 were included in the study. The database was redesigned in 2012 as a 20% national patient-level sample with nonrepresentative sampling across hospitals. As a result, data collected after 2011 are not representative of a particular region or state and were therefore excluded from the analysis. Hospitalizations of adult patients (age ≥18 years) with a diagnosis of CS were identified by International Classification of Diseases, Ninth Revision codes. The primary exposure of interest was care in a public reporting vs. a nonreporting state. Implementation of public reporting of outcomes for percutaneous interventions began in 1995 in New York and 2003 in Massachusetts. As such, these were considered public reporting states. A regional comparison within the Northeast region was performed with Connecticut, Maine, Maryland, Rhode Island, and Vermont serving as nonreporting states. Following previously accepted methodology, data from New Jersey and Pennsylvania were excluded from the analysis due to inconsistent reporting [13]. The main outcome of interest was the use of MCS. The secondary outcome was in-hospital mortality. The main outcome was identified by codes for intra-aortic balloon pump (IABP), percutaneous ventricular assist device including Impella (Abiomed Inc., Danvers, MA) and TandemHeart (CardiacAssist, Inc., Pittsburgh, PA), extracorporeal membrane oxygenation (ECMO), percutaneous cardiopulmonary support, and cardiopulmonary bypass (CPB).

Propensity score matching was performed given differences in baseline characteristics. Patients with the nearest propensity scores in the two groups (public reporting vs. nonreporting states) were matched using 1 : 1 scheme without replacement using the Greedy method. Matching variables included sex, race, insurance status, household income, use of PCI, ST-segment elevation myocardial infarction, and 23 NIS-reported comorbidities. Maximum propensity difference (caliper width) allowed was 0.05. Patients without matched observation were excluded. Weights were generated with NIS specified sample weights. “Proc Survey Logistic” function was used in SAS, and “SVY” function was used in STATA. Adjusted logistical regression models were subsequently created to generate odds ratios for propensity score-matched cohorts. Stata IC 12.0 (Stata-Corp) and SAS 9.3 (SAS Institute, Inc.) were used for statistical analysis.

## 3. Results

A total of 13043 hospitalizations for CS were included of which 9664 occurred in reporting and 3379 in nonreporting states. Baseline characteristics of patients in reporting and nonreporting states are presented in Table 1. Both groups shared a similar mean age, slight male predominance, and similar Charlson Comorbidity Index. AMI was present in 54% of cases in reporting and 56% of cases in nonreporting states. Patients in reporting states presented to large urban teaching hospitals more frequently than those in nonreporting states. Overall MCS use was 32.8% in this high-risk population. Patients in reporting states received MCS less frequently than patients in nonreporting states (31.9% vs. 35.4%; *p* < 0.01). IABP and CPB were the most common used forms of MCS.

**Figure 1:**
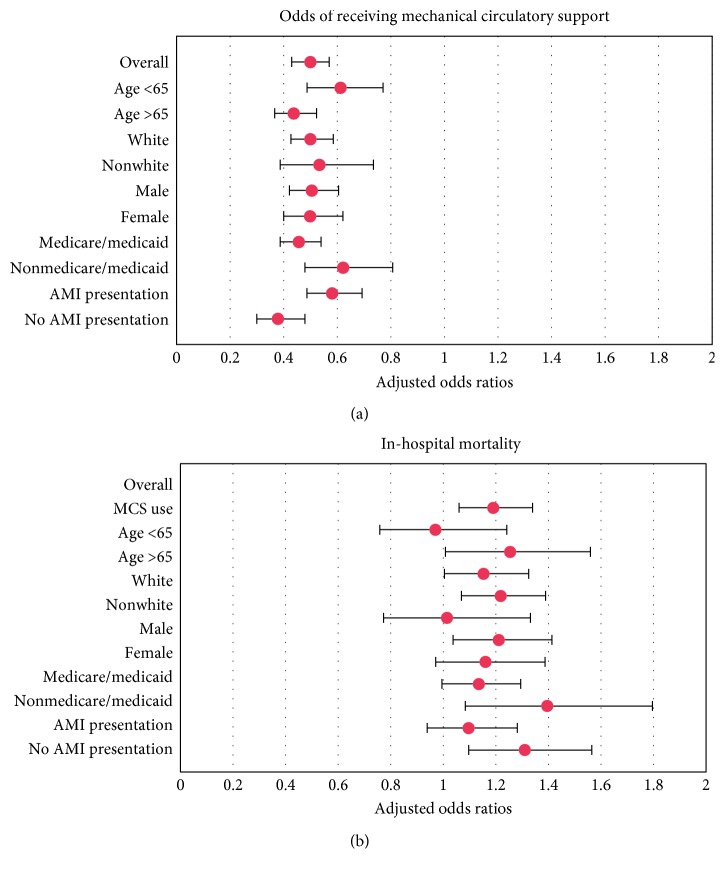
Use of mechanical circulatory support and in-hospital mortality for cardiogenic shock in reporting states. ^*∗*^AMI: acute myocardial infarction; MCS: mechanical circulatory support.

Table 2 presents baseline characteristics after 1 : 1 propensity score matching. MCS was used in 32.2% of cases in reporting and 36.4% of cases in nonreporting states (*p* < 0.01). After multivariate adjustment (Figure 1), MCS was used significantly less often in reporting states among all groups of patients. The effect was more pronounced among patients >65 years of age and those who did not present with AMI. Adjusted in-hospital mortality was higher in reporting states (OR 1.19; 95% CI 1.06–1.34; *p* < 0.01). The effect was more pronounced for patients who received MCS, those older than 65 years of age, and those who did not present with AMI.

**Figure 2:**
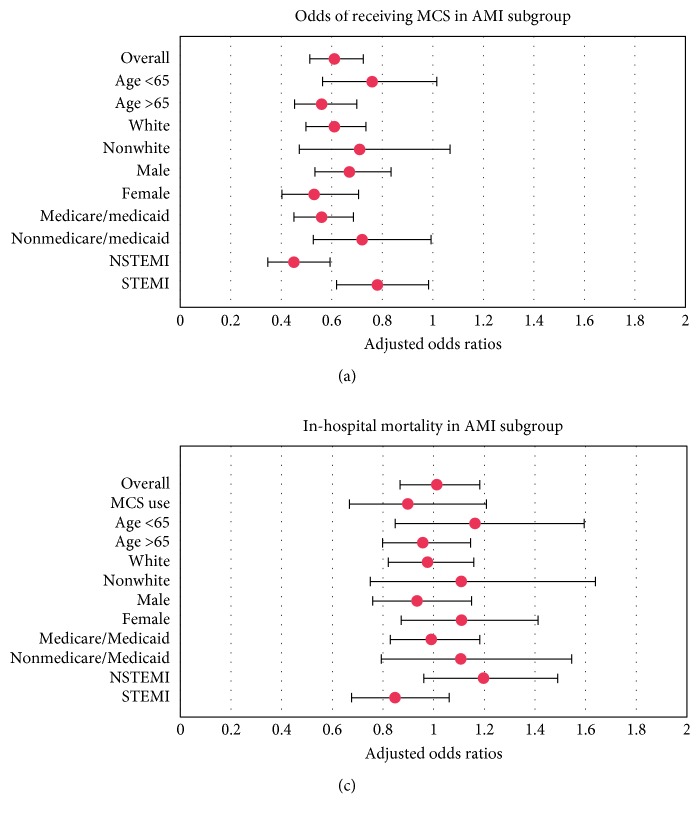
Use of mechanical circulatory support and in-hospital mortality in the subgroup of patients with acute myocardial infarction and cardiogenic shock in reporting states. ^*∗*^AMI: acute myocardial infarction; MCS: mechanical circulatory support; NSTEMI: non-ST-segment elevation myocardial infarction; STEMI: ST-segment elevation myocardial infarction.

Figure 2 presents the adjusted odds ratios for MCS use and in-hospital mortality for the subgroup of patients with AMI and CS. Odds of receiving MCS were lower in patients with AMI and CS in reporting states (OR 0.61; 95% CI 0.51–0.72; *p* < 0.01), regardless of type of myocardial infarction. There was no difference in adjusted odds ratio for in-hospital mortality in this subgroup (OR 1.01; 95% CI 0.87–1.18; *p*=0.87).

## 4. Discussion

This is the first study to analyze the impact of public reporting of outcomes on MCS utilization in patients with CS. Patients hospitalized in reporting states received MCS less frequently than patients in nonreporting states. Patients in reporting states had a higher in-hospital mortality. In the subgroup of patients with AMI and CS, MCS utilization was lower in reporting states regardless of the type of myocardial infarction, but in-hospital mortality was not significantly different.

A previous analysis of the NIS on the use of short-term MCS revealed that the use of nonpercutaneous forms of MCS increased by 101% from 2007 to 2011. Meanwhile, the use of percutaneous forms of MCS increased by 1,511% during the same period of time [14]. A more recent study of patients that received MCS for CS revealed similar increases in MCS use. Overall MCS utilization increased by 160% from 2004 to 2014. While IABP utilization continued to grow (140%), its relative use has declined compared to other forms of MCS (1421% for ECMO and 1229% for percutaneous devices) [15].

Public reporting of outcomes has been instrumental to improvements in healthcare, but concerns regarding unintended consequences remain. Risk-averse behaviors have been reported in physicians and hospitals caring for critically ill patients in states with public reporting [3, 6]. A regional analysis of the Medicare Provider Analysis and Review Files of patients with a diagnosis of AMI revealed that use of PCI was lower for patients in reporting states. The difference was more pronounced among patients with ST-segment elevation myocardial infarction, CS, and cardiac arrest [7]. An analysis of the NIS database from 2005 to 2011 revealed similar findings. In addition, in-hospital mortality was noted to be higher among patients in public reporting states [13].

Registry data have consistently suggested that public reporting may contribute to withholding of potentially life-saving interventions in critically ill patients. This has led some authors to suggest excluding the highest-risk patients from registry data. New York became the first state to exclude patients with CS and hypoxic encephalopathy from publicly reported PCI outcomes. Other states like Massachusetts offer risk-adjustment modifiers as an attempt to mitigate risk aversion [16]. However, risk-averse behavior has been reported even after changes in policy. A study of hospitalizations for AMI complicated by CS from 2002 to 2012 revealed that the exclusion of CS from public reporting in New York was followed by an increase in the use of PCI for patients with CS and a decrease in mortality. Nevertheless, PCI use remained low when compared to nonreporting states even after the change in policy [17]. One possible explanation is that patients with CS can be adjudicated post hoc as not “refractory” shock, which in turn places the provider at risk. Lower MCS utilization in public reporting states was noted in our study, presumably due to procedural risk aversion in this high-risk population. One possible explanation for the lower use of MCS in reporting states is that public reporting nudges physicians and institutions towards a more “conservative” approach in this very high-risk population. However, this hypothesis warrants further investigation.

Different strategies to reduce the impact of public reporting in the care for patients with CS have been proposed. A shift from procedure-based to disease-based outcomes reporting has been recommended to allow for accurate public reporting while reducing procedural risk aversion [16]. In addition, public reporting of institutional rather than individual operator outcomes may present a better option in patients presenting with CS or out-of-hospital cardiac arrest. Development of institutional protocols emphasizing early invasive hemodynamic monitoring and rapid initiation of MCS in patients presenting with AMI complicated by CS has resulted in promising initial results [9]. Institutions with systems in place to offer advanced therapies and with a proven record of improved outcomes could receive such patients preferentially which might ultimately enable the long-term goal of lowering mortality and improving outcomes in this very high-risk population.

### 4.1. Limitations

The NIS sampling design is statistically sound and has been used to estimate national healthcare trends. Strengths of the study include the heterogeneity of the population studied and large case volume. Nevertheless, some limitations are worth noting. First, given the code-based nature of the NIS database, the chance for miscoding or overcoding is present. Second, due to changes in the NIS design, data collected after 2011 are not representative of a particular state or region and as such were not included in the analysis. Risk-averse behavior has been noted to persist even after major policy changes, and thus we believe that the trends described here are likely to persist.

## 5. Conclusion

In conclusion, patients hospitalized for CS in public reporting states receive MCS less frequently than patients in nonreporting states. Further research is needed to optimize current reporting models and mitigate some of its unintended consequences.

## Figures and Tables

**Table 1:** Baseline characteristics of patients with cardiogenic shock, 2005–2011.

Variable	Public reporting (*N* = 9664)	Nonreporting (*N* = 3379)	*p* value
Age in years, mean ± SD	70.1 ± 0.5	69.5 ± 0.7	<0.01
Male	5479 (56.7)	1932 (57.2)	0.2
Race			<0.01
White	6987 (72.3)	2520 (74.6)	
Black	860 (8.9)	550 (16.3)	
Hispanic	599 (6.2)	37 (1.1)	
Other	879 (9.1)	162 (4.8)	
Missing	347 (3.6)	104 (3.1)	
Myocardial infarction presentation	5237 (54.2)	1875 (55.5)	<0.01
Charlson comorbidity index, mean ± SD	1.7 ± 0.01	1.7 ± 0.01	0.97
Obesity	531 (5.5)	405 (12)	<0.01
Hypertension	4484 (46.4)	1895 (56.1)	<0.01
Diabetes	2618 (27.1)	1145 (33.9)	<0.01
Peripheral vascular disease	850 (8.8)	547 (16.2)	<0.01
Chronic lung disease	2145 (22.2)	956 (28.3)	<0.01
Median household income category			<0.01
0–25th percentile	1845 (19.1)	520 (15.4)	
26–50th percentile	2010 (20.8)	598 (17.7)	
51–75th percentile	2077 (21.5)	976 (28.9)	
76–100th percentile	3324 (34.4)	1229 (36.4)	
Primary insurance			<0.01
Medicare	6426 (66.5)	2240 (66.3)	
Medicaid	869 (9)	243 (7.2)	
Private	1952 (20.2)	736 (21.8)	
Self-pay/no pay/others	415 (4.3)	148 (4.4)	
Hospital bed size			<0.01
Small	608 (6.3)	574 (17)	
Medium	1816 (18.8)	1280 (37.9)	
Large	7238 (74.9)	1520 (45)	
Hospital location and teaching status			<0.01
Rural	270 (2.8)	101 (3)	
Urban nonteaching	2058 (21.3)	1344 (39.8)	
Urban teaching	7334 (75.9)	1932 (57.2)	
Length of stay in days, median (IQR)	12 (7–21)	9 (5–17)	<0.01
Hospital charges in United States dollars, median (IQR)	41013 (21534–74680)	34833 (19538–60358)	<0.01
Discharge disposition			<0.01
Home	1130 (11.7)	527 (15.6)	
Transfer to short-term hospital	705 (7.3)	195 (5.8)	
Skilled nursing facility	2048 (21.2)	912 (27)	
Home healthcare	1391 (14.4)	378 (11.2)	
Mechanical circulatory support	3082 (31.9)	1196 (35.4)	<0.01
Percutaneous coronary intervention	1179 (12.2)	574 (17)	<0.01
Coronary artery bypass graft surgery	1009 (10.4)	616 (18.2)	<0.01
Aortic valve surgery	344 (3.6)	177 (5.2)	<0.01
Mitral valve surgery	377 (3.9)	143 (4.2)	0.39
In-hospital mortality	4348 (45)	1351 (40)	<0.01

Values reported as absolute numbers (percentage) unless otherwise specified. IQR = interquartile range.

**Table 2:** Propensity score matching.

Variable	Public reporting (*N* = 2982)	Nonreporting (*N* = 2982)	*p* value
Weighted numbers	14512	15498	
Age >65 years	1911 (64.1)	1994 (66.9)	0.02
Male	2212 (74.2)	1720 (57.7)	0.77
White	2218 (74.4)	2248 (75.4)	0.28
Myocardial infarction presentation	1657 (55.6)	1646 (55.2)	0.74
Medicare/Medicaid	2209 (74.1)	2191 (73.5)	0.58
Comorbid conditions			
Acquired immune deficiency syndrome	5 (0.2)	5 (0.2)	0.78
Collagen vascular disease	80 (2.7)	68 (2.3)	0.41
Congestive heart failure	623 (20.9)	626 (21)	0.87
Chronic pulmonary disease	802 (26.9)	796 (26.7)	0.91
Coagulopathy	584 (19.6)	611 (20.5)	0.38
Depression	169 (5.7)	164 (5.5)	0.69
Hypothyroidism	280 (9.4)	283 (9.5)	0.89
Liver disease	80 (2.7)	77 (2.6)	0.75
Lymphoma	35 (1.2)	29 (1)	0.54
Fluid/electrolyte disorders	1640 (55)	1657 (55.6)	0.66
Metastatic cancer	65 (2.2)	62 (2.1)	0.93
Neurological disorders	250 (8.4)	247 (8.3)	0.96
Obesity	298 (10)	271 (9.1)	0.24
Paralysis	83 (2.8)	77 (2.6)	0.69
Psychoses	71 (2.4)	68 (2.3)	0.73
Pulmonary circulation disorders	119 (4)	119 (4)	0.9
Solid tumor	62 (2.1)	65 (2.2)	0.93
Peptic ulcer disease	0 (0)	0 (0)	—
Valvular disease	253 (8.5)	250 (8.4)	0.82
Weight loss	217 (7.3)	229 (7.7)	0.59
Alcohol abuse	122 (4.1)	128 (4.3)	0.7
Drug abuse	74 (2.5)	65 (2.2)	0.44
Percutaneous coronary intervention	518 (17.4)	495 (16.6)	0.39
Coronary artery bypass graft surgery	300 (10.1)	553 (18.5)	<0.01
Aortic valve surgery	102 (3.4)	151 (5.1)	<0.01
Mitral valve surgery	101 (3.4)	124 (4.2)	0.11
Mechanical circulatory support	960 (32.2)	1085 (36.4)	<0.01

Values reported as absolute numbers (percentage) unless otherwise specified. *N* = number.

## Data Availability

Data used for this study were extracted from the publicly available National Inpatient Sample from the Healthcare Cost and Utilization Project.

## References

[1] Dehmer G. J., Drozda J. P., Brindis R. G. (2014). Public reporting of clinical quality data. *Journal of the American College of Cardiology*.

[2] Riley R. F., Don C. W., Aldea G. S. (2012). Recent trends in adherence to secondary prevention guidelines for patients undergoing coronary revascularization in Washington state: an analysis of the clinical outcomes assessment program (COAP) registry. *Journal of the American Heart Association*.

[3] Moscucci M., Eagle K. A., Share D. (2005). Public reporting and case selection for percutaneous coronary interventions: an analysis from two large multicenter percutaneous coronary intervention databases. *Journal of the American College of Cardiology*.

[4] Narins C. R., Dozier A. M., Ling F. S., Zareba W. (2005). The influence of public reporting of outcome data on medical decision making by physicians. *Archives of Internal Medicine*.

[5] Schneider E. C., Epstein A. M. (1996). Influence of cardiac-surgery performance reports on referral practices and access to care—A survey of cardiovascular specialists. *New England Journal of Medicine*.

[6] Apolito R. A., Greenberg M. A., Menegus M. A. (2008). Impact of the New York state cardiac surgery and percutaneous coronary intervention reporting system on the management of patients with acute myocardial infarction complicated by cardiogenic shock. *American Heart Journal*.

[7] Joynt K. E., Blumenthal D. M., Orav E. J., Resnic F. S., Jha A. K. (2012). Association of public reporting for percutaneous coronary intervention with utilization and outcomes among medicare beneficiaries with acute myocardial infarction. *JAMA*.

[8] van Diepen S., Katz J. N., Albert N. M. (2017). Contemporary management of cardiogenic shock: a scientific statement from the American heart association. *Circulation*.

[9] Basir M. B., Schreiber T., Dixon S. (2018). Feasibility of early mechanical circulatory support in acute myocardial infarction complicated by cardiogenic shock: the Detroit cardiogenic shock initiative. *Catheterization and Cardiovascular Interventions*.

[10] Basir M. B., Schreiber T. L., Grines C. L. (2017). Effect of early initiation of mechanical circulatory support on survival in cardiogenic shock. *The American Journal of Cardiology*.

[11] Singh V., Mendirichaga R., Savani G. T. (2017). Coronary revascularization for acute myocardial infarction in the HIV population. *Journal of Interventional Cardiology*.

[12] (NIS) HNIS (2011). *Healthcare Cost and Utilization Project (HCUP)*.

[13] Waldo S. W., McCabe J. M., O’Brien C., Kennedy K. F., Joynt K. E., Yeh R. W. (2015). Association between public reporting of outcomes with procedural management and mortality for patients with acute myocardial infarction. *Journal of the American College of Cardiology*.

[14] Stretch R., Sauer C. M., Yuh D. D., Bonde P. (2014). National trends in the utilization of short-term mechanical circulatory support: incidence, outcomes, and cost analysis. *Journal of the American College of Cardiology*.

[15] Strom J. B., Zhao Y., Shen C. (2018). National trends, predictors of use, and in-hospital outcomes in the mechanical circulatory support for cardiogenic shock. *EuroIntervention*.

[16] Wasfy J. H., Borden W. B., Secemsky E. A., McCabe J. M., Yeh R. W. (2015). Public reporting in cardiovascular medicine. *Circulation*.

[17] McCabe J. M., Waldo S. W., Kennedy K. F., Yeh R. W. (2016). Treatment and outcomes of acute myocardial infarction complicated by shock after public reporting policy changes in New York. *JAMA Cardiology*.

